# Physiological Chemistry

**Published:** 1906-01

**Authors:** John A. Miller

**Affiliations:** Buffalo. N. Y.


### Physiological Chemistry.

By JOHN A. MILLER, PH. D., Buffalo. N. Y.

SULPHUR AND PHOSPHORUS METABOLISM ON AN ABUNDANT PROTEID DIET by Karl Bornstein, (Pflüger's Arch. 106, 66; J. C. Soc., 88, 99.) The higher the percentage of neutral phosphorous and sulphur in the urine, the smaller is the oxidative power of the organism. The experiments recorded lend support to the doctrine that an abundant proteid diet improves the cellular activities of the organism.

---

ORGANIC PHOSPHORUS IN URINE by D. Symmers (J. Path. Bact. 10, 159; J. C. Soc., 88, 102.) The estimation of inorganic phosphates is not a true index of phosphorus metabolism; in various pathological conditions the phosphoric acid in organic combination may be frequently 25-50 per cent. of the total. The excretion of organic phosphorous is to a certain extent rhythmical. The amount is pronounced in lymphatic leucemia, and especially in degenerative nervous diseases.

It may be due to an increase in endogenous phosphorised katabolites, or may be an expression of lessened oxidation which normally would give inorganic phosphates as the end-products. The theory that it originates from bone is dismissed, for in extensive disease of bone, like osteomalacia, the output of phosphoric acid is not increased.

---

PEPTIC AND TRYPTIC DIGESTION OF PROTEIDS by D. Lawroff, (Zeil: Physiol. Chem. 43, 447; J. C. Soc., 88, 178.) The prolonged action of 0.5 per cent. hydrochloric acid leads to the formation, only more slowly, of the same products as those formed during peptic digestion. The experiments were mainly performed with gelatin and hemoglobin.

---

THE COMPOSITION OF CASEOUS DEPOSITS IN TUBERCLE by E. Schmoll (Chem. Centr. 1905, 280; J. C. Soc., 88, 272.) The chief material in tubercular caseous deposits is a coagulated proteid which in composition approaches albumin, globulin, and also fibrin. Any characteristic constituents of cell-nuclei are not detectable. Cholesterol and lecithin are also present.

---

UREA [IN FUNGI] by R. Gaze (Arch. Pharm. 243, 78; J. C. Soc., 88, 277.) Urea was isolated from the fungus *Lycoperdon Bovi-*

equal amounts from mature and immature specimens. From *Lycoperdon cervinum,* much mannitol could be isolated, but no urea.

---

INFLUENCE OF OZONE ON THE LUNGS by C. Bohr and V. Maar. (Chem. Centr. 1905, 1, 945; J. C. Soc., 88, 329.) Ozone causes injury to the lung tissue, increases the oxygen intake and lessens the carbon dioxide output.

---

CHEMISTRY OF CANCER, II. ABNORMAL FERMENTATIVE OCCURRENCES by Carl Neuberg (*Berlin Klin. Woch.,* 1905, 118; J. C. Soc., 88, 338.) The action of radium emanations on carcenomatous tissue is to influence the ferment action in the cancer cells. By autolysis of liver-cancer, a characteristic product appears, namely, free pentose; this is not formed from normal liver. This was confirmed in a case where the liver metastases originated from cancer of the stomach; the latter yielded no pentose. The high percentage of pentose in cancerous tissues is due to richness in nuclei. Normal liver juice does not influence the rate of autolysis in the lungs, but leads to the formation of albumoses; the juice of liver cancer leads to the opposite result, namely, an increase in simple cleavage products.

---

THE DISTRIBUTION OF NITROGEN IN THE URINE by G. Satta, (Beits. Chem. Physiol. Path. 6, 358; J. C. Soc., 88, 407.) In normal individuals taking no carbohydrates, the ammonia and purine nitrogen is increased at the expense of the other substances which are precipitable by phosphotungstic acid. In diabetes, there is always an increase in the monoamino-acid fraction; ammonia is increased, whilst the urea is correspondingly diminished. In a dog without a pancreas, the urea excretion remains normal, but the monoamino-acids increase.

---

ACTION OF FORMIC ACID ON TREMORS by E. Clément (Compt. Rend. 140, 1198; J. C. Soc., 88, 408.) The increase of muscular tonus said to be the result of administration of formic acid, led to its being given for tremors. It was given to a woman aged sixty-five who had trembled for ten years, and to a man aged seventy-two who had trembled for eighteen years. The result was amelioration of the condition.

---

ACTION OF CAMPHOR ON THE CIRCULATION by E. Seligmann (Arch. Exp. Path. Pharm. 53, 333; J. C. Soc., 88, 409.) Camphor in certain circumstances strengthens the heart-beat, and induces regular beats in a surviving heart which exhibits fibrillary contractions. No certain action on the vaso-motor mechanism could be determined.

